# Faulty evidence for superconductivity in ac magnetic susceptibility of sulfur hydride under pressure

**DOI:** 10.1093/nsr/nwac086

**Published:** 2022-05-10

**Authors:** J E Hirsch

**Affiliations:** Department of Physics, University of California, San Diego, USA

**This article is a comment on 'High-temperature superconductivity in sulfur hydride evidenced by alternating-current magnetic susceptibility' by Huang et al (https://doi.org/10.1093/nsr/nwz061)**.

**The response to this article is 'Reply: Faulty evidence for superconductivity in ac magnetic susceptibility of sulfur hydride under pressure' by Wang et al. (https://doi.org/10.1093/nsr/nwac087**.

Sulfur hydride was reported to become superconducting at high pressure and temperature [1]. Posteriorly, Huang and coworkers measured its ac magnetic susceptibility [2], reportedly confirming that finding. According to Semenok and Oganov [3], their work ‘sets a new standard for experimental studies of superconductivity at high pressure’. Instead I argue here that the ac susceptibility measurements of Huang *et al.* [2] provide no support for the existence of superconductivity in sulfur hydride. A more extended analysis is given in [4].

AC magnetic susceptibility is a superior test for superconductivity in materials under pressure [5]. A superconductor excludes magnetic flux, so upon cooling into the superconducting state a sharp drop in the ac magnetic susceptibility is observed. Because of the smallness of the sample required by the geometry of the diamond anvil cell, the detected signal is a small drop in a large signal arising from the superposition of the sample and the background magnetic responses. For that reason, it is customary to subtract from the total signal (the so-called ‘raw data’) the background signal, usually obtained by measuring the susceptibility at a lower pressure value such that no superconducting transition occurs in the temperature range of interest.

Huang *et al.* [2] reported ac magnetic susceptibility measurements for sulfur hydride for seven different pressure values. They plot in their Fig. 2 the data obtained after background subtraction for four different pressures.

The drops in the signals seen in their figure were interpreted as due to the onset of superconductivity [2]. In order to assess the validity and significance of these results, I requested from the authors the raw data and background signal measured. The authors kindly sent me these data and gave me additional details of the measurements upon request.

**Figure 1.**
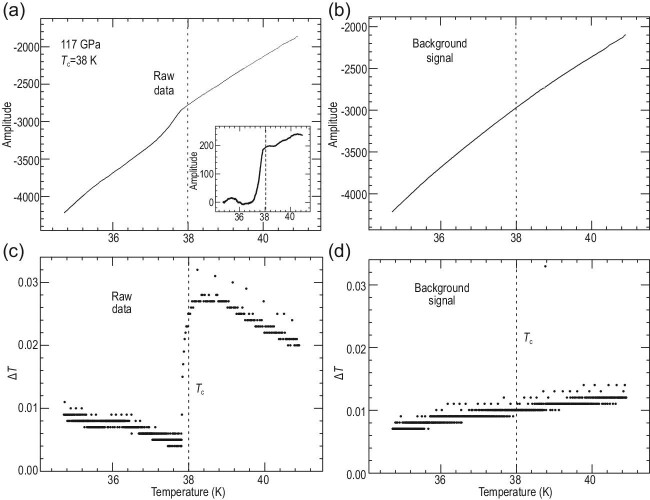
Raw data (a) and background signal (b) from which the susceptibility data shown in the inset were obtained. The lower panels show the change in temperature between subsequent measurements for the raw data (c) and background signal (d). The vertical dashed lines indicate the assumed transition temperature *T*_*c*_ = 38 K [2].

The top left and right panels of Fig. 1 show the raw data and background signal for one of the pressure values, 117 GPa. One can see in the raw data a break in

the slope and a faster drop below 38 K. When subtracting the background signal, the curve shown in the inset of the top left panel results, identical to the results reported in Fig. 2 of [2], appearing to indicate a superconducting transition.

However, when I plot Δ*T*, the difference in temperature between subsequent measurements, versus temperature, I obtain the lower left and right panels of Fig. 1. The background shows smooth behavior, but the raw data show a sharp break right below the assumed critical temperature *T*_c_ = 38 K, namely at *T* = 37.82 K.

I was informed that the system was being heated at a constant rate in the experiment. One may think that the sudden increase in the temperature step could indicate that the heat capacity of the system suddenly decreased. For a superconducting transition, this is in fact expected: the specific heat jump at the critical temperature is given by (*c*_*s*_ − *c*_*n*_)/*c*_*n*_ = 1.43, with *c*_*n*_ and *c*_*s*_ the heat capacities in the normal and superconducting states.

However, the temperature sensor cannot be placed in the diamond anvil cell next to the sample; that is physically impossible. Assuming that the temperature sensor is located at a distance *R* ∼ 1 cm from the sample, the temperature measured would correspond to that of a volume of order ∼10^7^ times larger than the volume of the sample, so it cannot possibly be influenced to the degree shown in the figure by a change in the heat capacity of the sample at the assumed transition temperature.

I found a similar anomaly in the raw data for the susceptibility measurements for pressure 130 GPa, for which the inferred superconducting transition temperature was 55 K. Data for 149 and 155 GPa also showed such anomalies, to a smaller degree, right at the assumed transition temperatures.

We have to conclude that the sudden changes in the temperature increments coinciding with the assumed critical temperatures are experimental artifacts. They imply that the observed changes in slope in the ac susceptibility observed at those assumed critical temperatures are a consequence of the same experimental artifacts and cannot be taken as evidence of superconducting transitions. There may have been unwanted/uncontrolled variations in the temperature steps, as well as discrepancies between what were the actual values of the temperature at the sample position versus what the thermometer measured at a different position, that account for these anomalies [4].

My findings here highlight the importance that authors make available their raw data for other scientists’ examination. Huang and coworkers did that exemplarily, as also did M. Debessai and coworkers recently for their ac susceptibility measurements of the element europium [6]. Upon examination of those raw data, both I [7] and the authors [8] concluded that the original conclusion that Eu becomes superconducting under pressure [6] is with high probability incorrect. In contrast, I have pointed out anomalous behavior of the ac susceptibility measurements [9] for the reported room-temperature superconductor C-S-H [10] and repeatedly requested the raw data for examination, but the corresponding author has declined to provide them [while this paper was being reviewed, part of the raw data (the measured voltage, but not the background signal) for C-S-H were provided by Dias and Salamat [11] ]. This attitude hinders the advancement of science.

The results discussed here, as well as recent analysis we [12,13] and others [14] have performed on other experimental data on hydride superconductors under pressure, indicate the urgent need for greater scrutiny of experimental data and lesser reliance on theoretical expectations in this field of research.
